# Genetic and clinical features of cerebellar ataxia with *RFC1* biallelic repeat expansions in Japan

**DOI:** 10.3389/fneur.2022.952493

**Published:** 2022-08-10

**Authors:** Masahiro Ando, Yujiro Higuchi, Junhui H. Yuan, Akiko Yoshimura, Shuntaro Higashi, Mika Takeuchi, Takahiro Hobara, Fumikazu Kojima, Yutaka Noguchi, Jun Takei, Yu Hiramatsu, Satoshi Nozuma, Yusuke Sakiyama, Akihiro Hashiguchi, Eiji Matsuura, Yuji Okamoto, Masahiro Nagai, Hiroshi Takashima

**Affiliations:** ^1^Department of Neurology and Geriatrics, Kagoshima University Graduate School of Medical and Dental Sciences, Kagoshima, Japan; ^2^School of Medicine, Faculty of Medicine, Kagoshima University, Kagoshima, Japan; ^3^Department of Physical Therapy, School of Health Sciences, Faculty of Medicine, Kagoshima University, Kagoshima, Japan; ^4^Department of Neurology and Clinical Pharmacology, Ehime University Hospital, Ehime, Japan

**Keywords:** cerebellar ataxia, *RFC1*, (AAGGG)exp, (ACAGG)exp, (AAAGG)_10−25_(AAGGG)exp

## Abstract

The recessive intronic pentanucleotide repeat AAGGG expansion of replication factor complex subunit 1 (*RFC1*) is associated with cerebellar ataxia, sensory neuropathy, and vestibular areflexia syndrome. And the clinical spectrum has been continuously expanding. We conducted this study to demonstrate the clinical and genetic features of a large-scale case series of Japanese patients with cerebellar ataxia with *RFC1* repeat expansions. We examined 1,289 Japanese patients with cerebellar ataxia and analyzed *RFC1* repeat expansions in 840 patients, excluding those with genetic diagnoses or an autosomal dominant inheritance pattern. For individuals where no product was obtained by flanking polymerase chain reaction (PCR), repeat-primed PCR was performed using primers specific for the following four repeat motifs: AAAAG, AAAGG, AAGGG, and ACAGG. *RFC1* analysis revealed multitype biallelic pathogenic repeat expansions in 15 patients, including (AAGGG)exp/(AAGGG)exp in seven patients, (ACAGG)exp/(ACAGG)exp in three patients, (AAGGG)exp/(ACAGG)exp in four patients, and (AAGGG)exp/(AAAGG)_15_(AAGGG)exp in one patient. Clinical analysis showed various combinations of cerebellar ataxia, vestibular dysfunction, neuropathy, cognitive decline, autonomic dysfunction, chronic cough, pyramidal tract disorder, parkinsonism, involuntary movement, and muscle fasciculation. Pathological *RFC1* repeat expansions account for 1.8% (15/840) of undiagnosed patients with cerebellar ataxia and sporadic/recessive/unclassified inheritance. Screening of *RFC1* repeat expansions should be considered in patients with cerebellar ataxia, irrespective of their subtype and onset age.

## Introduction

The recessive intronic pentanucleotide repeat AAGGG expansion in the replication factor complex subunit 1 (*RFC1*) gene was reported as a genetic basis of cerebellar ataxia, sensory neuropathy, and vestibular nerve palsy syndrome (CANVAS) in 2019 (1). Originally, *RFC1* repeat expansion was thought to be a common cause of late-onset ataxia (1), but thereafter, its clinical spectrum has expanded dramatically. Patients with *RFC1*-related disorders may develop varying degrees of cerebellar ataxia, neuropathy, and vestibular hypofunction, as well as in combination with other symptoms, such as pyramidal tract disorder, autonomic dysfunction, chronic cough, parkinsonism, involuntary movement, cognitive dysfunction, muscle fasciculation, and hyperCKemia (2–4).

Studies have reported that the carrier frequency of *RFC1* heterozygous (AAGGG)exp ranges from 0.7 to 4% in populations of northern European origin (1, 5, 6), with an estimated prevalence of *RFC1*-related disorders of 1:20,000–1:625. A similar allele frequency (2.24%) has been detected in the Chinese population (7). In Japan, heterozygous *RFC1* (AAGGG)exp has been found in 1/55 (1.8%) healthy control individuals (8). At this locus of *RFC1*, in addition to the reference allele (AAAAG)_11_, other nonpathogenic repeat motifs, (AAAAG)exp and (AAAGG)exp, have been reported (1). Regarding the pathogenic repeat motifs, other than (AAGGG)exp, (ACAGG)exp has been identified in the Asia-Pacific region (4, 9, 10) and (AAAGG)_10−25_(AAGGG)exp has been detected in Māori individuals (11).

In this study, we analyzed *RFC1* repeat expansions in a large case series of Japanese patients with cerebellar ataxia and describe the clinical and genetic features of 15 patients harboring biallelic *RFC1* pathogenic repeat expansions.

## Materials and methods

**Figure 1:**
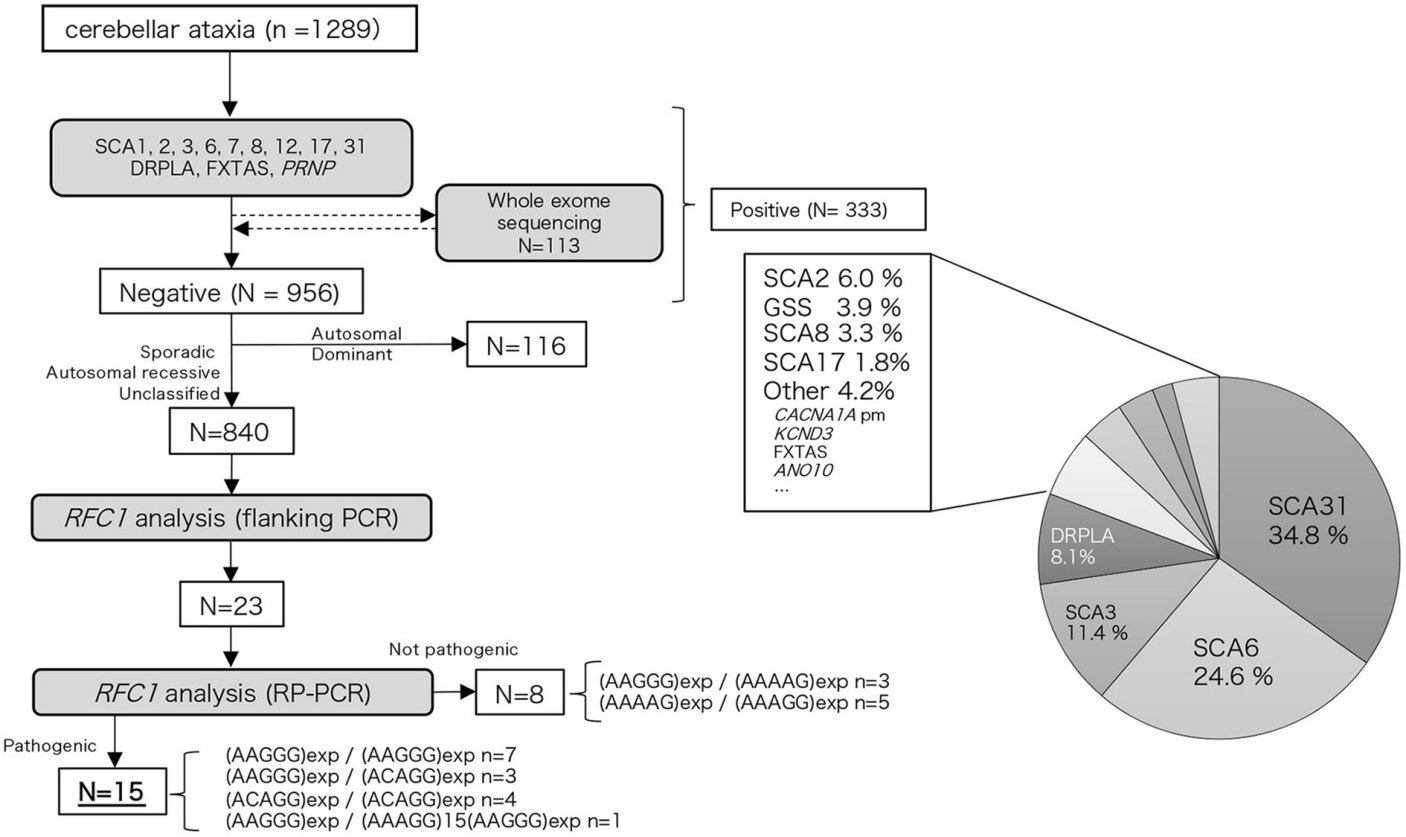
Flow chart of our study. We performed hereditary ataxia-related gene analysis for 1,289 cerebellar ataxia cases. Of the 956 cases that tested negative, 840 cases were analyzed for the *RFC1* repeat expansion.

We examined 1,289 Japanese patients with chronic progressive cerebellar ataxia and collected their blood samples from medical clinics/institutions in western Japan, primarily from the Kyushu region (Kagoshima, Miyazaki, Oita, Fukuoka, and Okinawa prefectures) and Ehime prefecture. Genomic DNA was extracted from peripheral blood or saliva using the Gentra Puregene Blood Kit (QIAGEN, Valencia, CA, USA) or the Oragene DNA self-collection kit (DNA Genotek, Ottawa, Ontario, Canada) according to the manufacturer's instructions. First, these samples were screened for repeat expansions associated with hereditary ataxia (SCA1, SCA2, SCA3, SCA6, SCA7, SCA8, SCA12, SCA17, SCA31, DRPLA, and FXTAS) and *PRNP*. The detailed procedure has been described elsewhere (12). Whole exome analysis using Ion Proton (ThermoFisher Scientific, Inc.) was conducted in part of the undiagnosed cases (*n* = 113, 11.7%); the detailed workflow has been described previously (13). A total of 333 patients were found to be positive for repeat expansions or mutations in hereditary ataxia-associated genes, including SCA31, SCA6, SCA3, DRPLA, SCA2, *PRNP*, SCA8, SCA17, *CACNA1A* (point mutation), *KCND3*, FXTAS, and *ANO10*. Among 956 undiagnosed patients, after excluding 116 patients with an autosomal dominant family history, *RFC1* analysis was conducted on 840 patients. The flowchart of our study is depicted in Figure 1. Among these patients, family history or consanguinity were found positive from 82 cases, and 678 cases were negative; 77 cases had no available family history data. One hundred and one cases were clinically diagnosed with multiple systems atrophy (MSA).

For individuals where no product was obtained by flanking PCR, repeat-primed PCR (RP-PCR) was performed using primers specific for the following four repeat motifs: AAAAG (benign), AAAGG (benign), AAGGG (pathogenic), and ACAGG (pathogenic) (1, 4, 9). The RP-PCR products were subjected to capillary electrophoresis using the ABI PRISM 3130xL Genetic Analyzer (Applied Biosystems, Foster City, CA, USA), and results were visualized using the Peak Scanner software (Applied Biosystems, Foster City, CA, USA).

The disease-associated haplotypes surrounding the *RFC1* repeat locus were determined using six common single nucleotide polymorphisms (SNPs; hg19), namely, rs899873773 (chr4:39334265), rs11939718 (chr4:39337634), rs13143570 (chr4:39355429), rs13124271 (chr4:39355434), rs13142220 (chr4:39355501), and rs368936934 (chr4:39357080).

The clinical data were obtained by review of the clinical notes in each medical clinics and institutions. Similarly autonomic function and vestibular function data was obtained from data retrospectively recorded in these clinical notes. In some patients, autonomic testing was undertaken using the Schellong protocol. Vestibular function was assessed during a general neurological examination and no patients had bedside head impulse test (HIT)/visually enhanced vestibulo-ocular reflex (VVOR)/dynamic visual acuity testing.

### Ethics statement

This study was approved by the Institutional Review Board of Kagoshima University. All patients and family members provided informed consent for participation in the study and genetic analysis.

## Results

### Genetic findings

Among the 840 patients with undiagnosed cerebellar ataxia in our case studies 23 patients did not have any product on the initial screening flanking PCR. Of these eight were found to have the known benign expansions—(AAGGG)exp/(AAAAG)exp *n* = 3, (AAAAG)exp/(AAAGG)exp *n* = 5 (Figure 1). The remaining 15 had no smear PCR product after RP-PCR of benign repeat motif [(AAAAG)exp or (AAAGG)exp], and the presence of a decremental sawtooth pattern on the Peak Scanner of the RP-PCR product for (AAGGG)exp or/and (ACAGG)exp. Among these 15 patients, biallelic pathological RFC1 repeat expansions comprised (AAGGG)exp/(AAGGG)exp in seven patients, (ACAGG)exp/(ACAGG)exp in three patients, (AAGGG)exp/(ACAGG)exp in four patients, and (AAGGG)exp/(AAAGG)15(AAGGG)exp in one patient.

**Figure 2:**
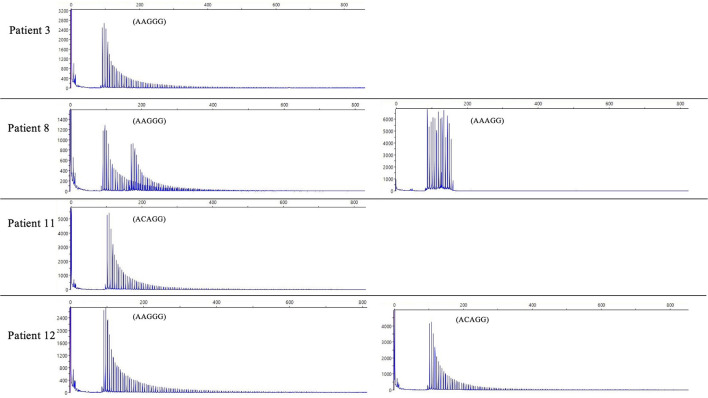
The result of RP-PCR. Sawtooth patterns on Peak Scanner of RP-PCR products, including (AAGGG)exp/(AAGGG)exp (patient 3), (AAGGG)exp/(AAAGG)_15_(AAGGG)exp (patient 8), (ACAGG)exp/(ACAGG)exp (patient 11), and (AAGGG)exp/(ACAGG)exp (patient 12).

We did not find any other inexplicable gaps within the RP-PCR saw tooth patterns and any repeat expansion which not explained by four repeats from our patients. The sawtooth patterns observed on the Peak Scanner of the RP-PCR product from each genotype are depicted in Figure 2. In the haplotype analysis, the 17.8-kb core haplotype was shared by all these 15 patients, including (AAGGG)exp, (ACAGG)exp, and (AAAGG)_15_(AAGGG)exp (Supplementary Figure 1).

### Clinical summary

The clinical and electrophysiological findings of the 15 patients with *RFC1* pathogenic repeat expansions are summarized in Tables 1, 2, respectively. Five patients had a family history or parental consanguinity. The average age of disease onset was 49.7 ± 17.3 (range 18–75) years. All patients had cerebellar ataxia. Sensory symptoms were observed in 10 patients, presenting with sensory disturbance (9/14, 64.3%) and abnormal sensory nerve conduction study (NCS; 7/9 77.8%). Motor symptoms were identified from four patients, showing muscle weakness/atrophy (4/13, 30.8%), or a slight decrease in CMAP or MNCV (3/9, 33.3%). Muscle fasciculations were observed in one patient (1/11, 9.1%). Serum creatine kinase level was within normal range in all patients with available data. Electrophysiological study was also carried out on three patients without neuropathic symptoms, and two showed no abnormal findings and one had subclinical sensory neuropathy. Overall, six patients had sensory-dominant neuropathy. One patient had normal NCS findings despite of having muscle weakness and sensory disturbance, possibly suggesting sensory and motor neuronopathy.

**Table 1:** Clinical features of patients with *RFC1* repeat expansions.

**Patient**	**Pt 1**	**Pt 2**	**Pt 3**	**Pt 4**	**Pt 5**	**Pt 6**	**Pt 7**	**Pt 8**	**Pt 9**	**Pt 10**	**Pt 11**	**Pt 12**	**Pt 13**	**Pt 14**	**Pt 15**
***RFC1* repeat**	**(AAGGG)exp**	**(AAGGG)exp**	**(AAGGG)exp**	**(AAGGG)exp**	**(AAGGG)exp**	**(AAGGG)exp**	**(AAGGG)exp**	**(AAGGG)exp**	**(ACAGG)exp**	**(ACAGG)exp**	**(ACAGG)exp**	**(AAGGG)exp**	**(AAGGG)exp**	**(AAGGG)exp**	**(AAGGG)exp**
	**(AAGGG)exp**	**(AAGGG)exp**	**(AAGGG)exp**	**(AAGGG)exp**	**(AAGGG)exp**	**(AAGGG)exp**	**(AAGGG)exp**	**(AAAGG)** **15(AAGGG)exp**	**(ACAGG)exp**	**(ACAGG)exp**	**(ACAGG)exp**	**(ACAGG)exp**	**(ACAGG)exp**	**(ACAGG)exp**	**(ACAGG)exp**
Exam age	51–55	76–80	21–25	66–70	21–25	76–80	31–35	51–55	61–65	71–75	76–80	76–80	66–70	66–70	71–75
Onset age	31–35	51–55	16–20	46–50	21–25	71–75	26–30	51–55	56–60	61–65	46–50	66–70	46–50	66–70	56–60
Family history or consanguinity	+	–	+	–	–	–	–	+	–	–	+	–	–	–	+
Cerebellar ataxia	+	+	+	+	+	+	+	+	+	+	+	+	+	+	+
Cerebellar atrophy	NA	+	+	+	+	+	–	–	+	+	+	+	+	+	+
Muscle weakness	NA	–	–	–	–	–	+	–	+	–	+	+	–	NA	–
Muscle atrophy	NA	–	–	–	–	–	+	–	+	–	NA	+	–	NA	–
Tendon reflex	NA	Absent	Hyper	Absent	Normal	Normal	Hyper	Normal	Normal	Normal	Decrease	Absent	Normal	Absent	Absent
Sensory disturbance	+	+	–	+	–	–	+	–	–	+	+	+	+	NA	+
NCS	Not done	SNp	Normal	SNp	Normal	Not done	Normal	SNp	Not done	Not done	SNp	SNp	Not done	Not done	SNp
Vestibular dysfunction	NA	–	–	–	–	NA	–	NA	NA	–	NA	–	NA	NA	–
Chronic cough	NA	–	–	–	–	NA	–	–	NA	–	–	–	NA	NA	–
Pyramidal sign	–	–	+	–	–	–	+	–	–	–	–	+pathological reflex	–	NA	–
Parkinsonism	–	–	–	–	–	–	–	–	–	–	–	–	–	NA	+
Cognitive impairment	NA	–	+HDS-R 25 MMSE 25	–	–	+HDS-R 25 MMSE 21	–	–	–	–	–	–	–	NA	–
Involuntary movement	NA	–	–	–	–	–	–	–	MyoclonusChorea	–	–	Myoclonus	–	NA	tremor
Autonimic dysfunction	NA	–	–	Constipation hypohidrosis	–	NA	–	–	NA	–	NA	–	Hypotension	NA	Neurogenic bladder Constipation
Muscle fasciculation	NA	–	–	–	–	NA	–	–	–	–	NA	Fasciculation	–	NA	–
Creatine kinase (IU/dL)	wnl	wnl	84	wnl	109	wnl	107	100	wnl	NA	86	143	–	NA	NA
Other			Low vision	SPECT; cerebellar hypoperfusion				SPECT; cerebellar hypoperfusion	cervical spondylosis	Sicca(SS-A/B Ab negative)		SPECT; cerebellar hypoperfusion			Datscan;reduced striatal uptake

*NCS: nerve conduction study; NA, not available; SNp, sensory neuropathy; HDS-R, Revised Hasegawa Dementia Scale; MMSE, Mini-Mental State Examination; wnl, within normal limit; SPECT, single-photon emission computed tomography*.

**Table 2:** Electrophysiological findings of 10 patients with *RFC1* pathogenic repeat expansions.

**Patient**	**Pt 2**	**Pt 3**	**Pt 4**	**Pt 5**	**Pt 7**	**Pt 8**	**Pt 11**	**Pt 12**	**Pt 15**
***RFC1*** **repeat**	**(AAGGG)exp**	**(AAGGG)exp**	**(AAGGG)exp**	**(AAGGG)exp**	**(AAGGG)exp**	**(AAGGG)exp**	**(ACAGG)exp**	**(AAGGG)exp**	**(AAGGG)exp**
	**(AAGGG)exp**	**(AAGGG)exp**	**(AAGGG)exp**	**(AAGGG)exp**	**(AAGGG)exp**	**(AAAGG)15** **(AAGGG)exp**	**(ACAGG)exp**	**(ACAGG)exp**	**(ACAGG)exp**
Median MNCV (m/s)	48	Normal	58.8	56.5	59.5	NA	49.4	50.2	50.9
MedianCMAP (mV)	5.5	Normal	8.2	11.5	8.2	NA	5.34	6.8	5
MedianSCV (m/s)	NE	Normal	40.2	45.2	58.3	Slow	NE	28.2	NE
MedianSNAP (μV)	NE	Normal	4.6	109	154	NA	NE	8.7	NE
TibialMNCV (m/s)	38.8	Normal	46	50.4	50.7	NA	37.9	52.2	38.9
TibialCMAP (mV)	16.8	Normal	17.2	10.3	16.5	NA	4.77	14.5	8.9
SuralSCV (m/s)	NE	Normal	32.5	49.1	49.6	NE	NE	29.7	NE
SuralSNAP (μV)	NE	Normal	1.8	18.2	14.2	NE	NE	10.5	NE

*MNCV, motor nerve conduction velocity; CMAP, compound motor action potential; SCV, sensory nerve conduction velocity; SNAP, sensory nerve action potential; NE, not evoked; NA, not available; Normal range: median CMAP > 3.1 mV; median MCV > 49.6 m/s; median SNAP > 7.0 μV; median SCV > 47.2 m/s; tibial CMAP > 4.4 mV; tibial MCV > 41.7 m/s; sural SNAP >5.0 μV; sural SCV > 40.8 m/s*.

Parkinsonism was observed in one patient (1/15 [6.7%]). Two patients showed a cognitive decline (2/13 [15.4%]). Involuntary movement were observed in three patients (3/13 [23.1%]). The evaluation of autonomic function was conducted via interviews revealed autonomic dysfunction from three patients (3/11 [27.3%]). One patient had hypotension, but she was not evaluated by the Schellong test. Three patients were evaluated using Schellong test, and none of them showed hypotension. None of the patients presented with obvious vestibular disturbance on general neurological examination, but no HIT/VVOR/dynamic visual acuity testing was performed.

### Radiological findings

**Figure 3:**
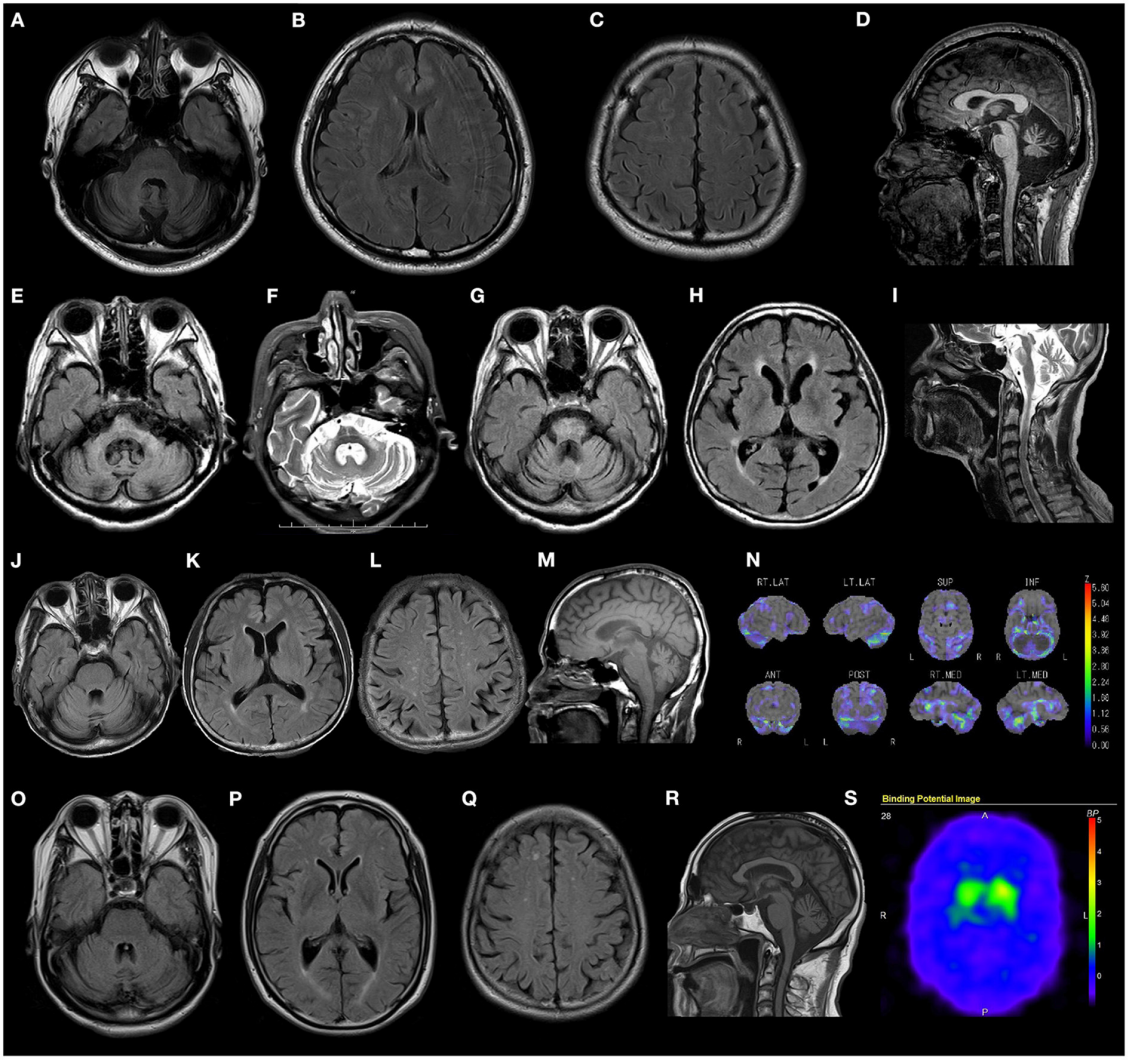
Radiological findings of patients with *RFC1* repeat expansions. **(A–D)** Brain MRI FLAIR images of patient 4 show marked cerebellar atrophy, without any atrophy in the brainstem and cerebrum. **(E–I)** Brain MRI FLAIR and T2 weighted images of patient 14 show marked cerebellar and brain stem atrophy, high T2 signal in the middle cerebellar peduncle, and hot cross bun sign. **(J–N)** Brain MRI FLAIR, T1 weighted images, and IMP-SPECT (decrease) images of patient 12 show slight cerebellar atrophy and decreased cerebellar blood flow, with no atrophy or hypoperfusion in the brainstem or cerebrum. **(O–S)** Brain MRI FLAIR images, T1 weighted images, and Datscan imaging of patient 15 show slight atrophy of the cerebellum and reduced striatal uptake of the dopamine transporter.

Most patients presented with cerebellar atrophy (12/14, 85.7%), and another patient without cerebellar atrophy showed cerebellar hypoperfusion revealed by single-photon emission computed tomography (SPECT). These patients exhibited varying degrees of cerebellar atrophy. The brain MRI findings of patient 12 revealed marked brainstem atrophy, high T2 signal in the middle cerebral peduncle, and hot cross bun sign. The IMP-SPECT findings of patient 14 showed decreased cerebellar blood flow, but the cerebral blood flow was normal. Datscan (^123^I-Ioflupane SPECT) of patient 15 with parkinsonism demonstrated reduced striatal uptake of the dopamine transporter. The brain MRI findings of patients 6, 12, 14, and 15; the IMP-SPECT findings of patient 12; and the Datscan of patient 15 are shown in Figure 3.

### Geographic distribution analysis

The origin of cases with biallelic *RFC1* pathogenic repeat expansions in our study was confirmed as follows: Kagoshima prefecture (seven patients), Okinawa prefecture (two patients), Ehime prefecture (two patients), and Oita/Miyazaki/Fukuoka/Shimane prefecture (one patient each). The geographic origin data of all the 840 patients and the 15 patients with biallelic *RFC1* pathogenic repeat expansions are summarized in Supplementary Figure 2. In regions where two or more cases were observed, there was no difference in the positive rate, suggesting that the *RFC1* repeat expansions are widely distributed in Japan.

## Discussion

Based on the analysis of *RFC1* repeat expansions of more than 800 Japanese patients with cerebellar ataxia, we identified pathogenic repeat expansions in 15 patients. We also demonstrated varied genotypes of *RFC1* repeat expansions, including (AAGGG)exp, (ACAGG)exp, and (AAAGG)_15_(AAGGG)exp.

The combination of cerebellar ataxia and bilateral vestibulopathy were first reported in 2004 and sensory neuropathy was found in three of the four cases described (14). Subsequently, a subgroup of patients with idiopathic bilateral vestibulopathy who also had cerebellar dysfunction and peripheral polyneuropathy was reported in 2007, suggesting a new syndrome (15). Thereafter, the condition was termed “cerebellar ataxia, vestibulopathy, neuropathy, vestibular areflexia syndrome (CANVAS)” in 2011, encompassing all their essential clinical features (16). In 2019, the *RFC1* biallelic AAGGG intronic repeat expansion was identified in patients with CANVAS with late-onset ataxia (1). Since then, the *RFC1* repeat expansion has been reported in various countries and multiple ethnic groups. Pathogenic repeat motifs, (ACAGG)exp and (AAAGG)_10−25_(AAGGG)exp, were reported in addition to the original repeat genotype (AAGGG)exp (4, 9–11). To date, biallelic (ACAGG)exp has been identified in one Japanese patient, one patient of Chinese descent residing in Indonesia, and one patient of Niue descent (4, 9). In an analysis conducted using the gnomAD dataset, the carrier frequency of (ACAGG)exp was 0% in Europe, 0.03% in Africa, and 0.26% in South Asia (4). The (ACAGG)exp allele was detected in seven patients in the present study, including (ACAGG)exp/(ACAGG)exp and (AAGGG)exp/(ACAGG)exp. These findings emphasize the need for screening (ACAGG)exp of *RFC1* in Asian patients, which differs from that in European patients. We also detected (AAAGG)_15_(AAGGG)exp in a Japanese patient with late-onset ataxia and subclinical sensory neuropathy or neuronopathy. This repeat genotype had been identified previously; our patient is the first patient of non-Māori ethnicity to be described with this variant and may represent a common ancestral origin given the history of Pacific migrations of the Polynesian peoples. (11, 17).

We conducted haplotype analysis using SNPs enrolled by a previous Japanese study (10). All the 15 patients shared a core haplotype (<17.8 kb), ranging from rs11939718 (chr4:39337634) to rs13142220 (chr4:39355501). This result was consistent with the results of another report on Japanese CANVAS cases (10). In that report rs368936934 was found to distinguish the (ACAGG)exp allele from the (AAGGG)exp allele with the C allele being associated with the former and the T with the latter. Such was the case for all our patients except patient 13 who carried the T allele in association with a (ACAGG) expansion. (10). In the 22.8-kb region analyzed in this study, the patient harboring (AAAGG)_15_(AAGGG)exp exhibited a slightly different haploblock compared with the patients harboring the other repeat motifs.

It is notable that our diagnostic yield of RFC1 repeat expansions was lower than that in other ataxia cohorts (1.8 vs. 3.2–22%) (1, 18–20). This may be accounted for by the inclusion of cases with MSA in our case series, and the predominant sporadic cases (80%) without family history and consanguinity. It is likely that some cases with non-hereditary ataxia, such as immune-mediated cerebellar ataxia, were mixed together.

We analyzed retrospectively, the clinical notes of the 15 patients with biallelic pathological RFC1 repeat expansions. In addition to cerebellar ataxia and neuropathy, which are the primary symptoms of CANVAS, we detected various combinations of cognitive decline, autonomic dysfunction, pyramidal tract disorder, parkinsonism, involuntary movement, and muscle fasciculation. None of our patients had vestibular dysfunction or chronic cough. It is likely that this is an underestimate given the pervasiveness of vestibular findings in other patients with progressive ataxia and older age seen in other case series (21). We acknowledge that the retrospective nature of our paper is a considerable drawback. HyperCKemia has been reported in patients with biallelic (ACAGG)exp (9, 10), but it was within the normal range in our patients. We analyzed the clinical symptoms of the following three genotypes: (AAGGG)exp/(AAGGG)exp, (ACAGG)exp/(ACAGG)exp, and (AAGGG)exp/(ACAGG)exp (Table 3). Cognitive impairment was detected only in the (AAGGG)exp/(AAGGG)exp genotype. Muscle weakness was observed in all three genotypes, but it was more common in the (ACAGG)exp/(ACAGG)exp genotype. Motor neuron involvement has been reported to be more pronounced in patients with (ACAGG)exp, (4, 10) which was consistent with our results (10). Involuntary movement and autonomic neuropathy were more common in patients with (AAGGG)exp/(ACAGG)exp. The mean ages of onset were 39.7 ± 20.4 years for patients with (AAGGG)exp/(AAGGG)exp, 56.7 ± 5.9 years for patients with (ACAGG)exp/(ACAGG)exp, and 61.5 ± 8.7 years for patients with (AAGGG)exp/(ACAGG)exp. In our analysis, patients with (AAGGG)exp/(AAGGG)exp tended to have a younger onset age than those with other repeat motifs, and one patient developing the disease at the age of 18. *RFC1*-related cerebellar ataxia has also been reported to occur at <10 years of age (11). Therefore, *RFC1* repeat analysis should be considered when necessary, irrespective of the age of onset.

**Table 3:** The proportion of clinical manifestations in each genotype.

***RFC1*** **repeat**	**(AAGGG)exp**	**(ACAGG)exp**	**(AAGGG)exp**	**(AAGGG)exp**	**All cases**
	**(AAGGG)exp**	**(ACAGG)exp**	**(ACAGG)exp**	**(AAAGG)15(AAGGG)exp**	
	***N*** **= 7**	***N*** **= 3**	***N*** **= 4**	***N*** **= 1**	***N*** **= 15**
Onset age	39.7 ± 20.4	56.7 ± 5.9	61.5 ± 8.7	51	49.7 ± 17.3
Cerebellar ataxia	7/7 [100%]	3/3 [100%]	4/4 [100%]	+	15/15 [100%]
Cerebellar atrophy	5/6 [83.3%]	3/3 [100%]	4/4 [100%]	–	12/14 [85.7%]
Muscle weakness	1/6 [16.7%]	2/3 [66.7%]	1/3 [33.3%]	–	4/13 [30.8%]
Muscle atrophy	1/6 [16.7%]	1/2 [50%]	1/3 [33.3%]	–	3/12 [25%]
hyporeflexia	2/6 [33.3%]	1/3 [33.3%]	3/4 [75%]	–	6/14 [42.9%]
Sensory disturbance	4/7 [57.1%]	2/3 [66.7%]	3/3 [100%]	–	9/14 [64.3%]
Vestibular dysfunction	0/5 [0%]	0/1 [0%]	0/2 [0%]	NA	0/8 [0%]
Choronic cough	0/5 [0%]	0/2 [0%]	0/2 [0%]	–	0/9 [0%]
Pyramidal sign	2/7 [28.6%]	0/3 [0%]	1/3 [33.3%]	–	3/14 [21.4%]
Parkinsonism	0/7 [0%]	0/3 [0%]	1/3 [33.3%]	–	1/14 [7.1%]
Cognitive impairment	2/6 [33.3%]	0/3 [0%]	0/3 [0%]	–	2/13 [15.4%]
Involuntary movement	0/6 [0%]	1/3 [33.3%]	2/3 [66.7%]	–	3/13 [23.1%]
Autonimic dysfunction	1/5 [20%]	0/1 [0%]	2/3 [66.7%]	–	3/10 [30%]
Muscle fasciculation	1/11 [9.3%]	1/11 [9.2%]	1/11 [9.1%]	1/11 [9.0%]	1/11 [9.1%]

*NA, not available*.

Regarding the radiological findings of our patients, the degree of cerebellar atrophy varied, with some patients showing severe cerebellar atrophy and others showing slight cerebellar atrophy with reduced cerebellar blood flow. The brain MRI findings of patient 14 demonstrated marked cerebellar atrophy and brainstem atrophy, high T2 signal in the middle cerebellar peduncle, and hot cross bun sign, features commonly associated with MSA. An association between biallelic RFC1 (AAGGG)exp and MSA-C has been reported in three Chinese patients (22). Patient 14 had cerebellar ataxia, but no Parkinsonism or autonomic neuropathy. She was clinically diagnosed with a high probability of MSA based on radiological findings. In addition, one of our patients presented with cerebellar ataxia and parkinsonism with reduced striatal uptake of the dopamine transporter. Visual compensation, cough and sensory symptom were discriminative factors against RFC1-negative patients (2). Our two patients with suspected MSA developed sensory disturbance and decreased tendon reflexes. Differential analysis between RFC1-related disorders and MSA may be difficult, and RFC1 analysis would be helpful in cases with neuropathic symptoms.

In conclusion, we describe multitype *RFC1* repeat expansions in 1.8% (15/840) of undiagnosed patients with cerebellar ataxia and sporadic/recessive/unclassified inheritance. These findings expand our understanding regarding the heterogeneity of *RFC1* repeat expansions and strengthen the necessity to perform relevant screening extensively in patients with cerebellar ataxia, irrespective of their phenotype or age of onset. Screening of ACAGG repeat expansions is indispensable, particularly in Asia.

## Data availability statement

The raw data supporting the conclusions of this article will be made available by the authors, without undue reservation.

## Ethics statement

The studies involving human participants were reviewed and approved by Institutional Review Board of Kagoshima University. The patients/participants provided their written informed consent to participate in this study.

## Author contributions

MA, YHig, JY, and HT contributed to the concept or design of the study. MA, YHig, JY, AY, and SH contributed to the analysis and interpretation of data. MA and HT produced the first draft of the manuscript. All authors provided input into subsequent drafts and reviewed and approved the final version for submission.

## Funding

This work was supported by Grants-in-Aid from the Research Committee of Ataxia, Health Labour Sciences Research Grant, the Ministry of Health, Labour and Welfare, Japan (201610002B). This research is also supported by the Research program for conquering intractable disease from Japan agency for Medical Research and development (AMED) (201442014A, 201442071A, 17929553) and JSPS KAKENHI Grant Numbers JP18H02742, JP20K16604, JP21K15702, and JP21H02842.

## Conflict of interest

The authors declare that the research was conducted in the absence of any commercial or financial relationships that could be construed as a potential conflict of interest.

## Publisher's note

All claims expressed in this article are solely those of the authors and do not necessarily represent those of their affiliated organizations, or those of the publisher, the editors and the reviewers. Any product that may be evaluated in this article, or claim that may be made by its manufacturer, is not guaranteed or endorsed by the publisher.
