# Supplementary material for: Genetic and clinical features of cerebellar ataxia with RFC1 biallelic repeat expansions in Japan
Source: Front Neurol. 2022 Aug 10;13:952493. doi: 10.3389/fneur.2022.952493 (PMC9404689; doi:10.3389/fneur.2022.952493)
Supplement: Supplementary file 1 [file Data_Sheet_1.docx]

Supplementary Material

# Supplementary Figures and Tables

## Supplementary Figures


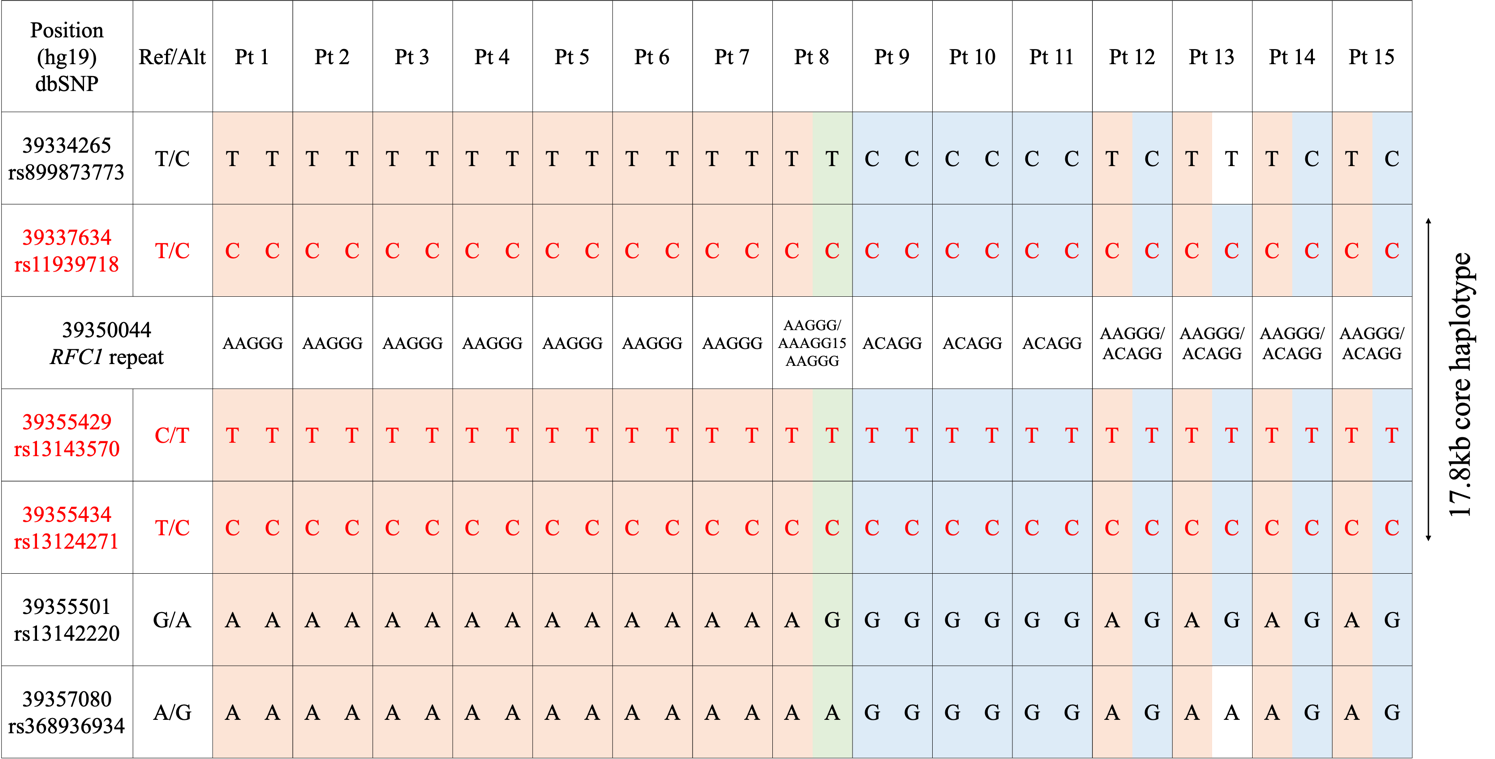


**Supplementary Figure 1.** Haplotype analysis of 15 patients carrying *RFC1* repeat expansions

Single nucleotide polymorphism (SNP)-based haplotype analysis reveals a 17.8 kb homologous block, shared by all of our 15 cases, regardless of the repeat expansion genotype. A longer homologous haplotype block of 22.8 kb (chr4: 39334265~39357080) was identified in all cases harboring (AAGGG)exp. Whereas patients with (ACAGG)exp share same SNP genotypes at rs899873773 (C/C), rs13142220 (G/G), and rs368936934 (G/G), except for patient 13. A distinguishable haplotype can be observed in patient 8 carrying (AAAGG)_15_(AAGGG)exp.


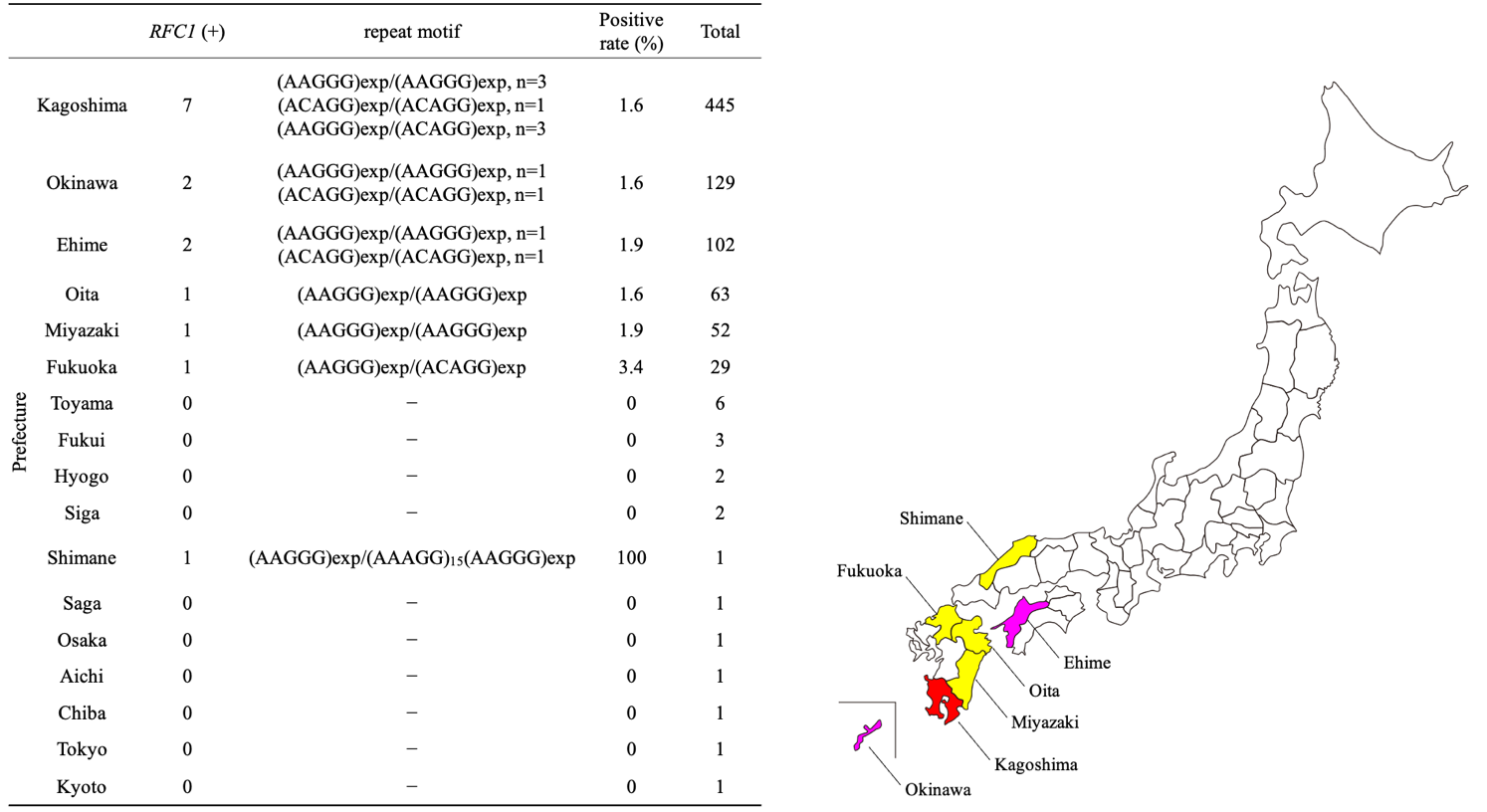


**Supplementary Figure 2.** Origin of our cases, positive rate, and repeat motif.

## Supplementary Table

**Supplementary Table 1.** Primer information
